# 5-methoxytryptophan ameliorates renal ischemia/reperfusion injury by alleviating endoplasmic reticulum stress-mediated apoptosis through the Nrf2/HO-1 pathway

**DOI:** 10.3389/fphar.2025.1506482

**Published:** 2025-04-14

**Authors:** Shaona Li, Hongjuan Yang, Bing Zhang, Lingyu Li, Xiangkun Li

**Affiliations:** ^1^ Department of Anesthesiology, The Affiliated Hospital of Qingdao University, Qingdao, China; ^2^ Department of Gynecology, The Affiliated Hospital of Qingdao University, Qingdao, China

**Keywords:** 5-methoxytryptophan, ischemia-reperfusion injury, endoplasmic reticulum stress, apoptosis, nuclear factor erythroid-2 related factor 2, heme oxygenase-1

## Abstract

**Background:**

Renal ischemia/reperfusion (I/R) injury is a prevalent clinical complication characterized by high incidence and mortality rates. The endogenous metabolite, 5-Methoxytryptophan (5-MTP), derived from tryptophan, possesses anti-inflammatory and antioxidant properties. However, its role in renal I/R injury remains unclear. In this study, we investigated whether 5-MTP could protect the kidney from I/R injury by ameliorating endoplasmic reticulum stress (ERS)-mediated apoptosis through the Nrf2/HO-1 pathway.

**Methods and results:**

We established models to examine renal I/R injury in C57BL/6J mice with bilateral renal pedicles clamped and HK-2 cells subjected to hypoxia/reoxygenation (H/R). The administration of 5-MTP improved renal tissue damage and kidney dysfunction impairment and reduced inflammation and oxidative stress. Moreover, 5-MTP attenuated ERS and ERS-mediated apoptosis, while upregulating Nrf2 and HO-1 expression. Additionally, Nrf2-deficient mice and cells were used to determine whether the Nrf2/HO-1 pathway was involved in the role of 5-MTP in alleviating ERS-mediated apoptosis. Nrf2 deficiency led to a partial reduction in the suppressive effects of 5-MTP on inflammation, oxidative stress, and ERS-mediated apoptosis.

**Conclusion:**

Our findings suggest that 5-MTP alleviates renal I/R injury by inhibiting ERS-related apoptosis via the Nrf2/HO-1 pathway.

## 1 Introduction

Renal ischemia/reperfusion (I/R) injury, characterized by an increase in morbidity and mortality rates, is a highly prevalent and clinically significant cause of acute kidney injury (Ma et al., 2021; Wilflingseder et al., 2020). Renal I/R injury often occurs as a secondary consequence of shock, myocardial infarction, kidney transplantation, and other conditions that may result in a reduction in renal blood flow (Scholz et al., 2021). Despite extensive efforts, clinicians find it difficult to effectively manage renal I/R injury. If not adequately controlled, renal I/R injury may progress to renal fibrosis, chronic kidney disease, or even uremia, thereby imposing a substantial burden on the global healthcare system (Dehnadi et al., 2017). Thus, effective new treatment approaches for renal I/R injury need to be developed urgently.

The apoptosis of renal tubular cells plays a key role in the occurrence and progression of renal I/R injury (Xia et al., 2023). Apoptosis, a nonlytic programmed cell death, is generally immunologically silent (Newton et al., 2024). Endoplasmic reticulum stress (ERS) is involved in the activation of apoptosis (Hong et al., 2021). ERS-mediated apoptosis is particularly important for the progression of renal I/R injury (Diaz-Bulnes et al., 2024). Thus, therapeutic strategies targeting ERS-mediated apoptosis have received considerable attention in research on renal I/R injury.

The endogenous metabolite, 5-Methoxytryptophan (5-MTP), derived from tryptophan, can be synthesized in various types of cells, including human fibroblasts and endothelial cells (Wu et al., 2020). Studies have found that 5-MTP possesses antitumorigenic, vascular-protective, and anti-fibrotic properties in renal disease (Fang et al., 2020). Additionally, 5-MTP has anti-inflammatory, antioxidative, and antipyroptotic effects on acute lung injury (Ma et al., 2023). However, the role of 5-MTP in modulating ERS-induced apoptosis during renal I/R injury is not clear.

Nrf2 is a prominent transcription factor characterized by its antioxidant and anti-inflammatory properties, and it is expressed in nearly all organs and tissues (Dinkova-Kostova and Copple, 2023). Under various pathological conditions, Nrf2 translocates to the nucleus, activating downstream genes in response to the cellular stimuli induced by inflammation and oxidative stress. This mechanism plays a crucial role in maintaining cellular homeostasis and strongly affects cell survival (Torrente and DeNicola, 2022). As one of the most important downstream factors of Nrf2, heme oxygenase-1 (HO-1) regulates Golgi stress, mitochondrial dynamics, and macrophage polarization (Lee et al., 2024). In another study, we found that the Nrf2/HO-1 pathway is involved in regulating ERS in renal I/R injury, ultimately improving renal outcomes (Li et al., 2023).

In this study, we assessed the ability of 5-MTP to alleviate renal I/R injury by mitigating ERS-mediated apoptosis. The involvement of the Nrf2/HO-1 pathway was also determined in Nrf2-deficient mice and cells.

## 2 Materials and methods

### 2.1 Animal model

C57BL/6J mice, weighing 22–25 g and aged 7–8 weeks, were obtained from Jinan Pengyue Laboratory Animal Breeding Co., Ltd. Nrf2-knockout (Nrf2-KO) mice with a C57BL/6J background were purchased from Jiangsu Huachuang Sino Pharma Co. The pentobarbital-isoflurane anesthesia protocol was used in our animal experiments. To establish the I/R injury model, atraumatic vascular clips were used to clamp the bilateral renal pedicles of mice for 30 min, followed by reperfusion for 24 h (Lee et al., 2020). After euthanasia, blood and renal tissues were collected immediately for further analysis.

To assess the renoprotective effect of 5-MTP, the mice were randomly assigned to four groups (*n* = 5). In the Sham and 5-MTP groups, renal pedicles were exposed but not subjected to clamping. The mice in the designed groups were administered an intraperitoneal injection (100 mg/kg) of 5-MTP (M4001, Sigma, United States) 30 min before the operation. The mice in the I/R group received an equal volume of saline. Additionally, to examine the association between the Nrf2/HO-1 pathway and the renoprotective effects of 5-MTP, wild-type (WT) or Nrf2-KO mice were randomized into three groups, each containing five mice.

### 2.2 Renal histology and function

Renal tissues were embedded in paraffin, cut into slices (4-μm-thick), and stained with hematoxylin-eosin (HE). Two experienced pathologists independently evaluated histopathological changes in the renal tissues. As described in previous studies, the tubular injury score, which ranges from 0 to 4, is used to assess the severity of renal tissue damage (Yan et al., 2020).

After collecting blood samples from the mice, the samples were left undisturbed at room temperature for 20 min. Next, the samples were centrifuged at 2,500 revolutions per minute (rpm)for 15 min at 4°C. The upper layer of serum was removed and stored at −20°C. The concentrations of blood urea nitrogen (BUN) and serum creatinine (SCr) were measured using an automated biochemical analyzer for animals (BS-240Vet, Mindray, China), which served as indicators to evaluate renal function in the mice.

### 2.3 Measurement of cytokines

The concentrations of IL-1β and IL-6 in mice serum were estimated using commercial ELISA kits (Solarbio, China). Following the manufacturer’s guidelines, absorbance readings were obtained at 450 nm and 630 nm, and the concentrations were subsequently calculated based on standard curves.

### 2.4 Assays of the glutathione/oxidized glutathione (GSH/GSSG) ratio and T-AOC

The total and oxidized glutathione levels were evaluated using a GSSG/GSH quantification kit (Jiancheng, China). The GSH/GSSG ratio was calculated. The total antioxidant capacity (T-AOC) was assessed by a T-AOC assay kit that uses the rapid ABTS method (Beyotime, China).

### 2.5 Immunofluorescence (IF) staining

The expression level of CCAAT/enhancer-binding protein (C/EBP) homologous protein (CHOP) was assessed by IF staining. After fixation with paraformaldehyde, tissue sections were washed three times with phosphate-buffered saline (PBS) and incubated overnight with an anti-CHOP antibody (CST #2895, United States). Next, the sections were incubated with a fluorescently labeled secondary antibody for 1 h. After incubation, 4,6-diamidino-2-phenylindole (DAPI) was used to visualize nuclei. IF intensity was quantitatively analyzed using the ImageJ software (NIH, United States).

### 2.6 TUNEL assay

TUNEL staining was conducted using a TUNEL Kit (Roche, United States). First, the sections were deparaffinized and treated with proteinase K for 10 min. Then, TUNEL labeling buffer was added. These sections were subsequently maintained in a humidified, light-tight container at 37°C for 1 h. The cell nuclei were stained with hematoxylin for 10 min.

### 2.7 Western blotting (WB) assay

The Nuclear and Cytoplasmic Protein Extraction Kit (Beyotime, China) was used for nuclear and cytoplasmic proteins extraction. Total proteins were extracted using a total protein isolation kit (Beyotime, China). Protein concentrations were assessed using a bicinchoninic acid (BCA) assay kit (Beyotime, China). SDS-PAGE (10% or 12%) was performed to separate the proteins. After electrophoresis, the separated proteins were transferred to polyvinylidene difluoride membranes. The primary antibodies against ATF4 (1:1,000, CST #11815, United States), ATF6 (1:1,000, CST #65880, United States), CHOP (1:1,000, CST #2895, United States), DR5 (1:1,000, Bioss, China), eIF2α (1:1000, Affinity AF6087, United States), HO-1 (1:2,000, CST #43966, United States), IRE1α (1:1000, Affinity DF7709, United States), Nrf2 (1:1,000, CST #12721, United States), p-IRE1α (1:1,000, Affinity AF7150, United States), PERK (1:1,000, Affinity AF5304, United States), p-eIF2α (1:1,000, Affinity AF3087, United States), p-PERK (1:1,000, Affinity DF7576, United States), β-actin (1:1,000, Affinity AF7018, United States) and GAPDH (1:1,000, Affinity AF7021, United States) were incubated overnight. The corresponding secondary antibodies were subsequently incubated for 1 h, and the blots were analyzed using the ImageJ software (NIH, United States).

### 2.8 Quantitative real-time PCR (qRT–PCR) analysis

RNA was extracted using TRIzol reagent (Invitrogen, United States). The PrimeScript RT Master Mix (Takara, Japan) was used to synthesize cDNA. The qRT-PCR procedure was performed using SYBR Green qPCR master mix (Takara, Japan); the primer sequences used are listed in Supplementary Table S1.

### 2.9 Bioinformatics analysis

We obtained the GSE212678 dataset from the GEO database and subsequently identified the differentially expressed genes (DEGs) between I/R and sham mice (Huang et al., 2024). GO and KEGG enrichment analyses were performed on the DEGs (Supplementary Table S2). We obtained Structure Data File (SDF) and Simplified Molecular Input Line Entry System (SMILES) files for the 5-MTP from PubChem (https://pubchem.ncbi.nlm.nih.gov/). The therapeutic targets of 5-MTP in renal I/R injury were predicted using the SwissTargetPrediction (Daina et al., 2019), PharmMapper (Wang et al., 2017), BindingDB (https://www.bindingdb.org), and GeneCards (https://www.genecards.org/) databases. A protein-protein interaction (PPI) network was constructed using the STRING database (https://www.string-db.org/), followed by visualization with Cytoscape (v3.9.0). Additionally, the key factors were identified with the cytoHubba in Cytoscape.

### 2.10 Cell treatment

HK-2 cells were obtained from the Cell Bank of the Chinese Academy of Sciences. DMEM and fetal bovine serum were purchased from Gibco (United States). A hypoxic environment was mimicked using a three-gas incubator with 1% O_2_, 94% N_2_, and 5% CO_2,_ and the cells were maintained in a hypoxic environment for 24 h. Next, the cells were cultured with a fresh medium under normoxic conditions for 6 h. Before exposure to hypoxia/reoxygenation (H/R) treatment, the cells were treated with various doses of 5-MTP dissolved in dimethyl sulfoxide (DMSO) for 12 h. The cells were then divided into five groups.

### 2.11 RNA interference

Custom-designed Nrf2 siRNA and negative control (NC) siRNA were purchased from Shanghai GenePharma Co., Ltd. (China). The cells were efficiently transfected with these siRNAs using Lipofectamine 3000 (Invitrogen, United States). The qRT-PCR analysis was conducted to assess the efficacy of knocking down Nrf2 after transfection for 24 h.

### 2.12 Cell viability

To assess the viability of HK-2 cells subjected to H/R, the Cell Counting Kit-8 (CCK-8) assay (Beyotime, China) was performed. The cells were transferred to a 96-well plate. Each well received 10 μL of CCK8 solution and was incubated for 2.5 h at standard temperature. The absorbance was recorded at 450 nm.

### 2.13 Apoptosis assay

The cell apoptosis rate was assessed using an Annexin V-FITC/PI Apoptosis Kit (Elabscience, China). HK-2 cells were seeded at a density of 1 × 10^5^ cells/well in six-well plates. Following incubation for 24 h, modeling and drug administration were conducted as previously described, and the cells were subsequently collected for further procedures. After centrifugation, the cells were rinsed and resuspended in a binding buffer. Next, the cells were incubated with Annexin V-FITC and PI reagents for 15 min. Finally, flow cytometry was performed using a BD flow cytometer (United States).

### 2.14 Statistical analysis

The data are presented as the mean ± standard deviation. The differences among and between groups were determined by one-way or two-way ANOVA with *post hoc* Bonferroni test using the Graph Prism 9 software (GraphPad Software, United States). All differences were considered to be statistically significant at P < 0.05.

## 3 Results

### 3.1 5-MTP protected the kidney from I/R injury *in vivo*

**FIGURE 1:**
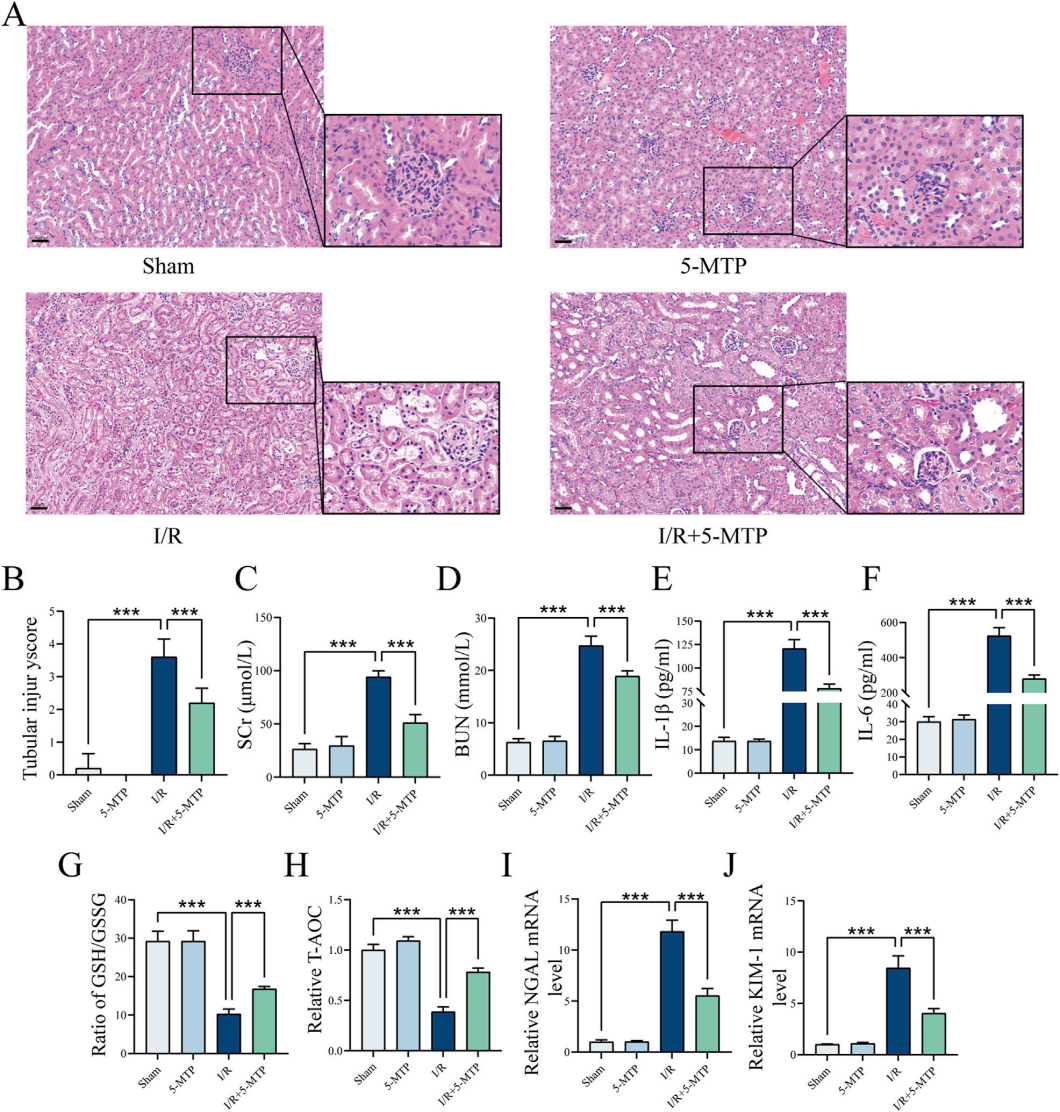
5-MTP protected the kidney from I/R injury *in vivo*. **(A)** HE staining was performed to evaluate renal histopathological alterations induced by I/R injury (×200, scale bar: 50 μm). **(B)** Two pathologists independently assessed tubule injury scores in a double-blinded manner, using HE staining as the basis for evaluation. **(C,D)** Serum SCr and BUN levels. **(E,F)** Serum IL-1β and IL-6 levels. **(G,H)** The GSH/GSSG ratio and T-AOC were assessed. **(I,J)** The mRNA expression levels of NGAL and KIM-1. The data are presented as the mean ± SD and assessed by conducting one-way ANOVA and the Bonferroni correction for multiple comparisons, *n =* 5 for each group; *P < 0.05, **P < 0.01, and ***P < 0.001.

To evaluate the renoprotective effect of 5-MTP pretreatment on renal I/R injury, histopathological analysis was performed, and the tubular injury score was assessed by HE staining. Compared to the Sham group, the I/R group presented severe pathological damage, specifically characterized by tubular necrosis, dilatation, edema, inflammatory infiltration, and cast formation. Notably, 5-MTP pretreatment significantly attenuated these pathological changes (Figure 1A). Similarly, the tubular injury score was considerably higher in the I/R group than in the Sham group. This score was considerably lower in the I/R+5-MTP group than in the I/R group (Figure 1B).

Renal function was assessed by detecting the BUN and SCr levels (Figures 1C,D). The I/R group presented higher BUN and SCr levels, indicating impaired renal function. However, 5-MTP pretreatment substantially improved renal function. To further investigate the level of inflammation, we measured the IL-1β and IL-6 levels (Figures 1E,F). I/R injury significantly increased the serum IL-1β and IL-6 levels; however, 5-MTP suppressed the secretion of these inflammatory cytokines. Additionally, to evaluate the levels of oxidative stress in the kidney, we detected the GSH/GSSG ratio and T-AOC (Figures 1G,H). I/R injury reduced the GSH/GSSG ratio and T-AOC, indicating an increase in oxidative stress, which was mitigated by 5-MTP pretreatment. We also detected the mRNA expression levels of Neutrophil Gelatinase-Associated Lipocalin (NGAL) and Kidney Injury Molecule-1 (KIM-1), which are markers of renal injury, by conducting qRT-PCR (Figures 1I,J). I/R injury significantly increased NGAL and KIM-1 mRNA levels, indicating renal proximal tubular damage. However, 5-MTP pretreatment significantly attenuated these changes. Our findings indicated that 5-MTP pretreatment exerts renoprotective effects, particularly by alleviating renal tissue injury, improving renal function, and mitigating inflammatory responses and oxidative stress.

### 3.2 5-MTP mitigated ERS-mediated apoptosis in mice exposed to renal I/R injury

**FIGURE 2:**
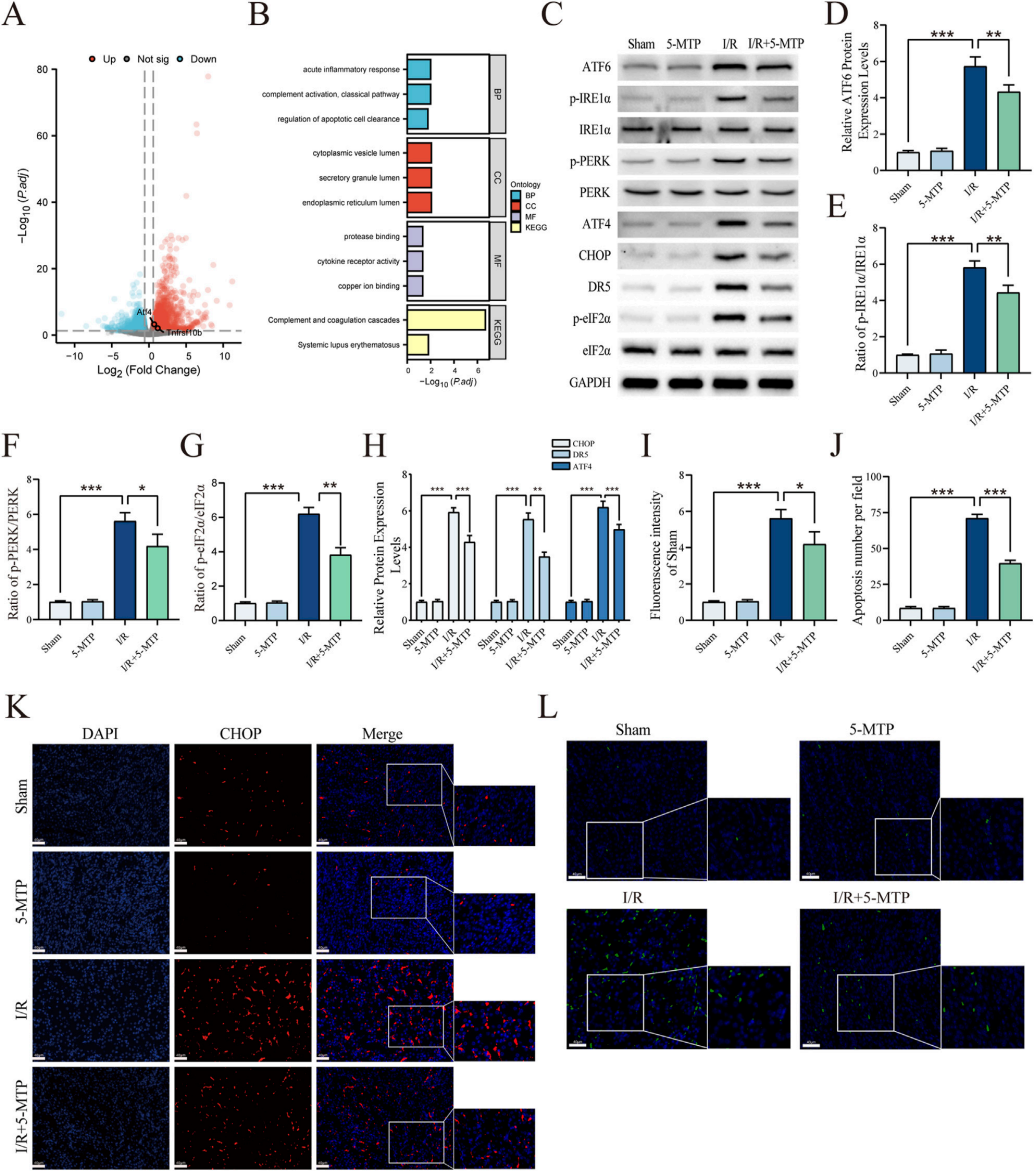
5-MTP mitigated ERS-mediated apoptosis in mice exposed to renal I/R injury. **(A)** The DEGs between renal I/R injury and sham mice (GSE212678). The dotted line indicates the threshold for DEGs, with blue and red dots representing genes with low and high expression in renal I/R injury mice, respectively. **(B)** GO and KEGG enrichment analyses of DEGs. **(C)** Representative images of WB assays are shown. **(D)** The relative protein expression level of ATF6 was determined via normalization to that of GAPDH, and the Sham group was set as the baseline (value of 1). **(E–G)** The relative phosphorylation levels of PERK, IRE1α, and eIF2α were detected by measuring the ratio of phosphorylated to total proteins, and the Sham group was set as the baseline (value of 1). **(H)** The relative levels of expression of ERS-mediated apoptosis proteins (ATF4, CHOP, and DR5) in kidney tissues were evaluated and normalized to those of GAPDH, and the Sham group was set as the baseline (value of 1). **(I)** Relative fluorescence intensity of CHOP (red). **(J)** Quantification of the TUNEL staining results. **(K)** CHOP expression in renal tissues was visualized by conducting IF staining (×400, scale bar: 40 μm). **(L)** Representative TUNEL staining (green) of renal tissues (×400, scale bar: 40 μm). The data are presented as the mean ± SD and evaluated by conducting one-way ANOVA and the Bonferroni correction for multiple comparisons, *n =* 5 for each group; *P < 0.05, **P < 0.01, and ***P < 0.001.

To evaluate the level of ERS-mediated apoptosis in renal I/R injury, we initially used bioinformatics methods. Specifically, the DEGs between sham and renal I/R mice in the GSE212678 dataset were analyzed and 3042 DEGs were identified, which included 1,713 upregulated and 1,329 downregulated genes (|log_2_FC| > 0.58, *P*.adj < 0.05) (Figure 2A). The mRNA expression levels of ATF4 and DR5, which are both crucial factors in ERS-mediated apoptosis, increased significantly, indicating the activation of ERS-mediated apoptosis in I/R model mice. By conducting GO and KEGG analyses, we found that the DEGs are involved primarily in the regulation of apoptotic cell clearance and the endoplasmic reticulum lumen (Figure 2B). To elucidate the results of the bioinformatics analysis, WB assays were performed to determine whether 5-MTP attenuated ERS and ERS-mediated apoptosis caused by renal I/R injury (Figures 2C–H). We assessed the level of ERS by determining the expression levels of ATF6, p-PERK, PERK, IRE1α and p-IRE1α through WB analysis. These results indicated that I/R injury considerably increased the protein expression level of ATF6 and the phosphorylation levels of PERK and IRE1α. However, pretreatment with 5-MTP partially decreased this effect. Moreover, renal I/R injury markedly increased the expression of ATF4, CHOP, and DR5, along with eIF2α phosphorylation level, whereas 5-MTP mitigated these changes. The IF results revealed that I/R injury greatly increased CHOP expression, whereas 5-MTP mitigated this change (Figures 2I,K). Apoptosis plays a key role in exacerbating various diseases caused by ERS (Zhang et al., 2022a). Apoptosis in kidney tissues was assessed by TUNEL staining (Figures 2J,L). The I/R group presented a significantly greater number of TUNEL-positive cells. Notably, 5-MTP greatly alleviated apoptosis in the I/R group. These findings indicates that 5-MTP can alleviate ERS and ERS-mediated apoptosis in mice with I/R.

### 3.3 5-MTP activated the Nrf2/HO-1 pathway in mice during renal I/R injury

**FIGURE 3:**
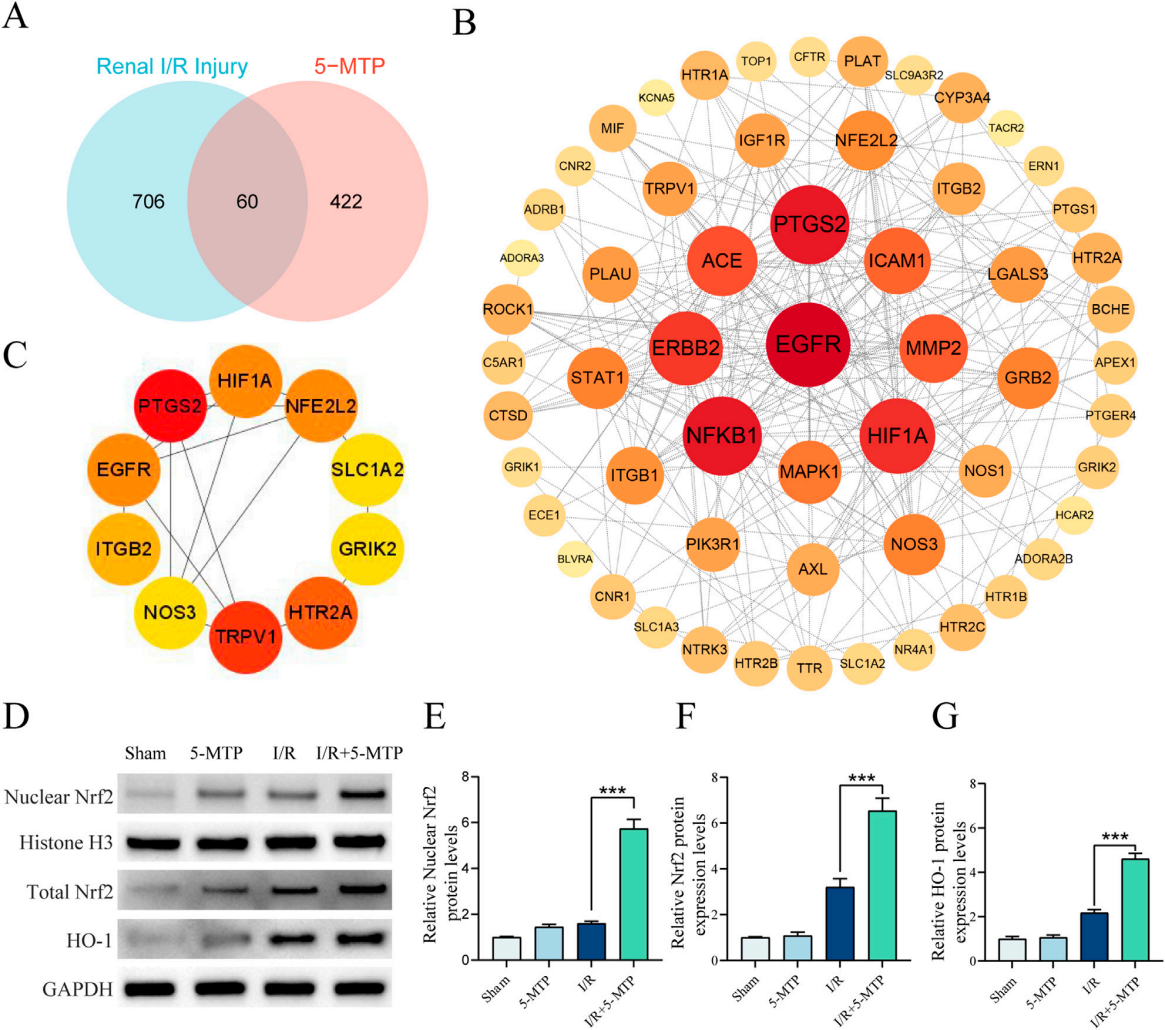
5-MTP activated the Nrf2/HO-1 pathway during renal I/R injury. **(A)** Venn diagram illustrating potential targets of 5-MTP in renal I/R injury. **(B)** PPI network analysis was conducted to demonstrate the interrelationships between these proteins. The color scale and size of the nodes indicate the degree of connectivity. Redder and larger nodes have a higher degree of connectivity, indicating that a protein in the network had greater importance. **(C)** The core targets (NFE2L2, HIF1A, PTGS2, EGFR, ITGB2, NOS3, TRPV1, HTR2A, GRIK2, and SLC1A2) were identified using the BottleNeck algorithm in Cytohubba. **(D)** Representative images of WB assays are shown. **(E)** The relative nuclear protein expression levels of Nrf2 were determined by normalization to the level of Histone H3, and the Sham group was set as the baseline (value of 1). **(F,G)** The relative protein expression levels of total Nrf2 and HO-1 were determined by normalization to the level of GAPDH, and the Sham group was set as the baseline (value of 1). The data are presented as the mean ± SD and evaluated by conducting one-way ANOVA and the Bonferroni correction for multiple comparisons, *n =* 5 for each group; *P < 0.05, **P < 0.01, and ***P < 0.001.

To investigate the molecular mechanisms underlying the renoprotective effect of 5-MTP, the SwissTargetPrediction, PharmMapper, and BindingDB databases were used to identify 482 potential targets of 5-MTP (13 duplicate targets eliminated). Next, genes related to renal I/R injury were retrieved from the GeneCards database, and Venn diagram analysis was performed to identify 60 overlapping genes (Figure 3A). To elucidate the primary target of 5-MTP, a PPI network was constructed using the STRING database (Figure 3B). The BottleNeck algorithm in Cytohubba was used to evaluate the correlations among the targets (Figure 3C). We found that *NFE2L2* exhibited a strong correlation. Another study revealed that 5-MTP can enhance acute lung injury recovery by activating the Nrf2/HO-1 pathway (Ma et al., 2023). We performed WB analysis to confirm the activation of the Nrf2/HO-1 pathway by 5-MTP in renal I/R injury (Figures 3D–G). The results revealed that 5-MTP increases both total Nrf2 expression levels and nuclear translocation of Nrf2, accompanied by an elevation in HO-1 protein expression. These findings confirm 5-MTP can activate the Nrf2/HO-1 pathway in renal I/R injury.

### 3.4 5-MTP alleviated H/R-induced injury in HK-2 cells by suppressing ERS-mediated apoptosis through the Nrf2/HO-1 pathway

**FIGURE 4:**
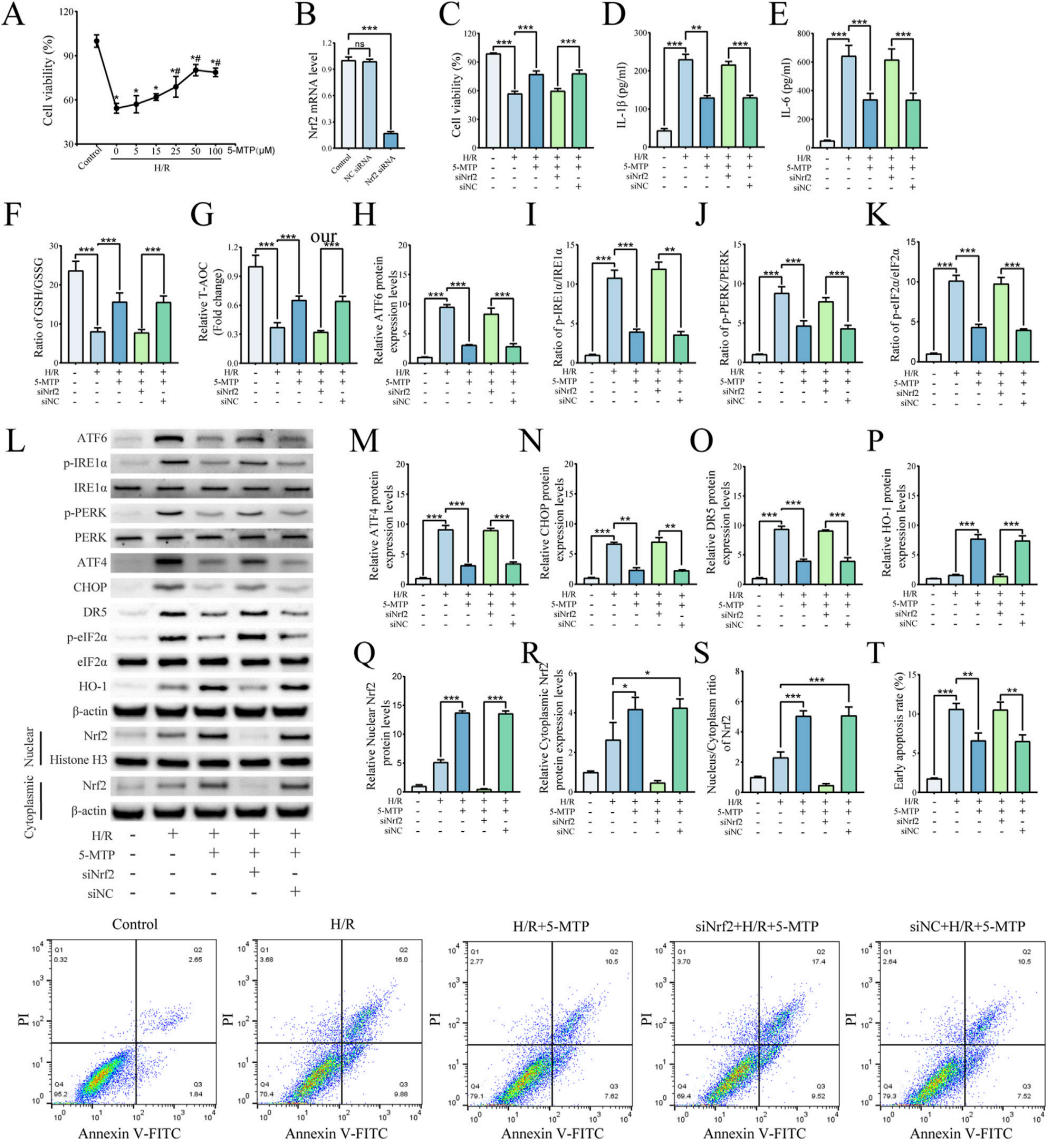
5-MTP alleviated H/R-induced injury in HK-2 cells by suppressing ERS-mediated apoptosis through the Nrf2/HO-1 pathway. **(A)** HK-2 cell viability following pretreatment with various concentrations of 5-MTP before H/R was evaluated by CCK-8 assays. *Significant difference from the control group (*P* < 0.05). #Significant difference from the H/R group (*P* < 0.05). **(B)** Relative Nrf2 mRNA levels were determined by qRT-PCR. **(C)** Cell viability was compared among different groups of HK-2 cells. **(D,E)** Levels of IL-1β and IL-6. **(F,G)** The GSH/GSSG ratio and T-AOC levels. **(H)** The relative protein expression level of ATF6 was determined via normalization to that of β-actin, and the control group was set as the baseline (value of 1). **(I–K)** The relative phosphorylation levels of PERK, IRE1α, and eIF2α were detected by measuring the ratio of phosphorylated to total proteins, and the control group was set as the baseline (value of 1). **(L)** Representative images of Western blotting assays. **(M–O)** The relative expression levels of ERS-mediated apoptosis proteins (ATF4, CHOP, and DR5) were evaluated and normalized to those of β-actin, and the control group was set as the baseline (value of 1). **(P)** Relative protein expression levels of HO-1 were determined via normalization to the level of β-actin, and the control group was set as the baseline (value of 1). **(Q)** The relative nuclear Nrf2 protein levels were determined by normalization to the level of Histone H3, and the control group was set as the baseline (value of 1). **(R)** The relative cytoplasm Nrf2 protein levels were determined by normalization to the level of cytoplasm β-actin, and the control group was set as the baseline (value of 1). **(S)** The relative nucleus-to-cytoplasm ratios of Nrf2 were analyzed, and the control group was set as the baseline (value of 1). **(T)** The rates of early apoptosis were detected via flow cytometry. The data are presented as the mean ± SD and evaluated by conducting one-way ANOVA and the Bonferroni correction for multiple comparisons, *n =* 3 for each group; *P < 0.05, **P < 0.01, and ***P < 0.001.

The Nrf2 gene was silenced in HK2 cells via siRNA. Using these Nrf2-knockdown cells, we confirmed the association between the Nrf2/HO-1 pathway and the amelioration of H/R injury by 5-MTP. The CCK-8 assay was conducted to assess the viability of HK-2 cells subjected to H/R and the effect of 5-MTP treatment at different concentrations. Before H/R exposure, the cells were incubated with 5, 15, 25, 50, or 100 μM 5-MTP for 12 h. Dose-dependent protection was observed at 5–50 μM (Figure 4A). Additionally, no significant difference in cell viability was observed between the doses of 100 and 50 μM (*P* > 0.05). Therefore, 50 μM 5-MTP was selected for use in subsequent experiments. The efficacy of Nrf2 knockdown was conformed by qRT-PCR (Figure 4B). As shown in Figure 4C, 5-MTP could ameliorate the damage caused by H/R. However, this cytoprotective effect of 5-MTP was notably inhibited in Nrf2-silenced cells. To assess the anti-inflammatory effect of 5-MTP *in vitro*, we measured the IL-1β and IL-6 levels. We found that 5-MTP significantly decreased the concentrations of both cytokines. However, silencing Nrf2 inhibited the anti-inflammatory effects of 5-MTP (Figures 4D,E). Additionally, based on the T-AOC levels and the GSH/GSSG ratio, 5-MTP showed the ability to mitigate oxidative stress, whereas knocking down Nrf2 significantly attenuated the antioxidant effect of 5-MTP (Figures 4F,G). As shown in Figures 4P–S, treatment with 5-MTP significantly increased the nuclear-to-cytoplasmic ratio of Nrf2, supporting active nuclear translocation. Concurrently, cytoplasmic Nrf2 levels exhibited a moderate elevation, suggesting that 5-MTP may also enhance total Nrf2 protein stability or synthesis. These findings collectively demonstrate that 5-MTP activates the Nrf2/HO-1 pathway through a dual mechanism involving nuclear translocation and potential modulation of Nrf2 protein levels. Consistent with Nrf2 activation, 5-MTP treatment robustly increased HO-1 expression. To evaluate the role of 5-MTP in ERS-mediated apoptosis *in vitro*, we conducted WB analysis to examine the expression levels of proteins related to ERS and ERS-mediated apoptosis (Figures 4H–O). H/R significantly increased the levels of ATF6, ATF4, CHOP, and DR5, as well as the levels of phosphorylated PERK, IRE1α, and eIF2α. However, 5-MTP effectively reversed this change, indicating its ability to mitigate H/R-induced ERS-mediated apoptosis. This effect of 5-MTP was not observed in the siNrf2+H/R+5-MTP group. Flow cytometry was then conducted to assess the early apoptosis, and the results revealed that 5-MTP significantly inhibited H/R-induced apoptosis (Figure 4T). However, this effect was also significantly inhibited in cells in which Nrf2 was knocked down. Our *in vitro* studies reveal that 5-MTP can mitigate ERS-mediated apoptosis by activating the Nrf2/HO-1 pathway.

### 3.5 The renoprotective effects of 5-MTP was attenuated in Nrf2-KO mice

**FIGURE 5:**
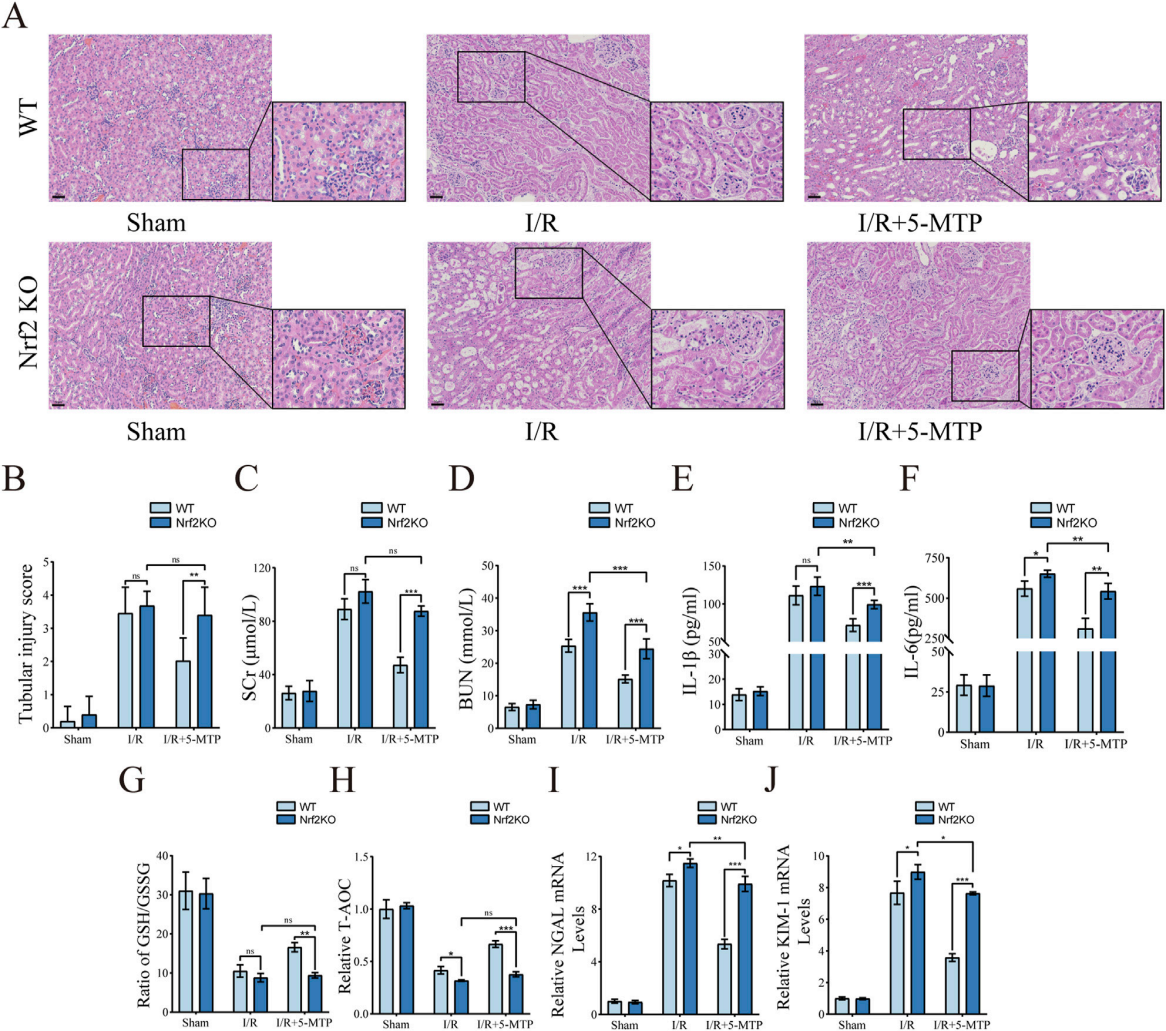
The renoprotective effects of 5-MTP was attenuated in Nrf2-KO mice. **(A)** HE staining was used to evaluate renal histopathological alterations induced by I/R injury (×200, Scale bar: 50 μm). **(B)** Two pathologists independently assessed tubule injury scores in a double-blind manner, utilizing HE staining as the basis for evaluation. **(C,D)** The serum SCr and BUN levels. **(E,F)** The serum IL-1β and IL-6 levels. **(G,H)** The GSH/GSSG ratio and T-AOC levels were assessed. **(I,J)** The mRNA expression levels of NGAL and KIM-1. The data are presented as the mean ± SD using two-way ANOVA and the Bonferroni test for multiple comparisons, *n* = 5 for each group. ^ns^ *P* > 0.05, *P < 0.05, **P < 0.01, ***P < 0.001.

To evaluate the role of the Nrf2/HO-1 pathway in mediating the renoprotective effects of 5-MTP *in vivo*, we compared outcomes between WT and Nrf2-KO mice. Compared to WT mice, Nrf2-KO mice subjected to I/R and 5-MTP presented more severe renal injury and much higher tubular injury scores (Figures 5A,B). By assessing SCr and BUN, we found that the improvement in renal function decline caused by 5-MTP in renal I/R injury was substantially suppressed in Nrf2-KO mice (Figures 5C,D). We also assessed whether the anti-inflammatory and antioxidant abilities were suppressed in Nrf2-KO mice (Figures 5E–H). Compared to WT mice, Nrf2-KO mice subjected to I/R and 5-MTP presented markedly higher levels of IL-1β and IL-6 and significantly lower GSH/GSSG ratios and T-AOC levels. Moreover, a considerable increase in NGAL and KIM-1 mRNAs indicated a significant attenuation of the efficacy of 5-MTP in mitigating kidney injury in Nrf2-KO mice (Figures 5I,J). These results suggest that the therapeutic efficacy of 5-MTP in I/R injury is strongly associated with the Nrf2/HO-1 pathway.

### 3.6 The suppression of ERS-mediated apoptosis by 5-MTP was abolished in Nrf2-KO mice

**FIGURE 6:**
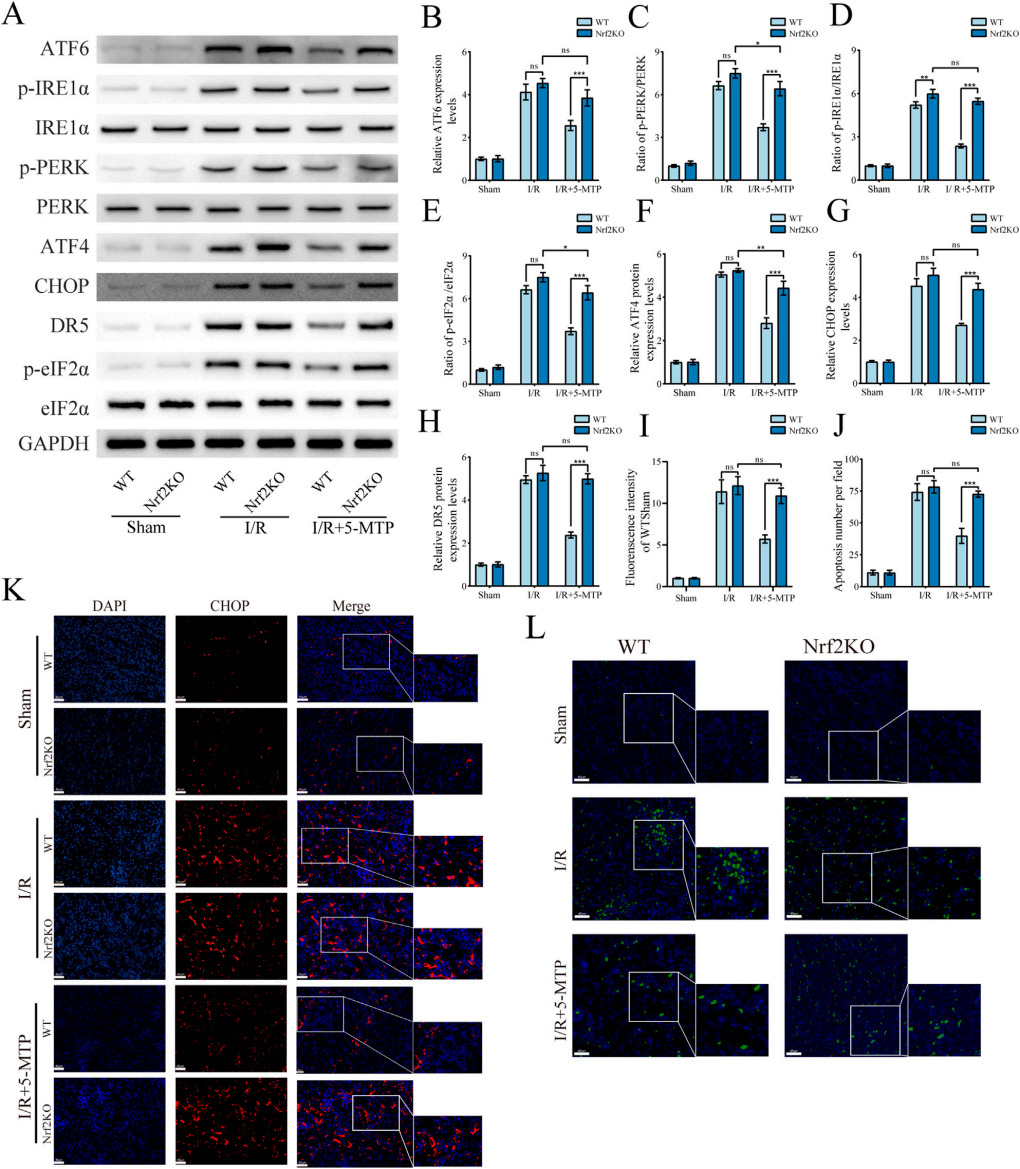
The suppression of ERS-mediated apoptosis by 5-MTP was abolished in Nrf2-KO mice. **(A)** Representative images of WB assays are shown. **(B)** The relative protein expression level of ATF6 was determined via normalization to that of GAPDH, and the WT + Sham group was set as the baseline (value of 1). **(C–E)** The relative phosphorylation levels of PERK, IRE1α, and eIF2α were detected by measuring the ratio of phosphorylated proteins to total proteins, and the WT + Sham group was set as the baseline (value of 1). **(F,H)** The relative expression levels of ERS-mediated apoptosis proteins (ATF4, CHOP, and DR5) were evaluated and normalized to those of GAPDH, and the WT + Sham group was set as the baseline (value of 1). **(I)** The fluorescence intensity of CHOP (red). **(J)** Quantification of the TUNEL staining results. **(K)** CHOP expression in renal tissues was visualized by conducting IF staining (×400, scale bar: 40 μm). **(L)** Representative TUNEL staining (green) of renal tissues (×400, scale bar: 40 μm). The data are presented as the mean ± SD and evaluated by conducting two-way ANOVA and the Bonferroni correction for multiple comparisons; *n =* 5 for each group; ^ns^ *P* > 0.05, *P < 0.05, **P < 0.01, and ***P < 0.001.

We subsequently assessed the expression of relevant proteins *in vivo* to confirm the role of the Nrf2/HO-1 pathway in the process by which 5-MTP alleviates ER stress-mediated apoptosis. The results of the WB analysis revealed considerably higher expression levels of ATF6 and higher phosphorylation of PERK and IRE1α in the Nrf2KO+I/R+5-MTP group compared to WT controls (Figures 6A–D). Concurrently, proteins associated with ERS-mediated apoptosis (ATF4, CHOP, and DR5) and the phosphorylation of eIF2α increased significantly in Nrf2-KO mice (Figures 6E–H). To validate these findings, we performed IF staining to evaluate CHOP protein levels (Figures 6I,K). The Nrf2KO+I/R+5-MTP group presented higher CHOP expression than the WT+I/R+5-MTP group. Additionally, renal tissue apoptosis was evaluated via TUNEL staining (Figures 6J,L). The Nrf2KO+I/R+5-MTP group presented a higher number of TUNEL-positive cells. These findings suggest that 5-MTP mitigates ERS-mediated apoptosis through the activation of the Nrf2/HO-1 pathway.

## 4 Discussion

**FIGURE 7:**
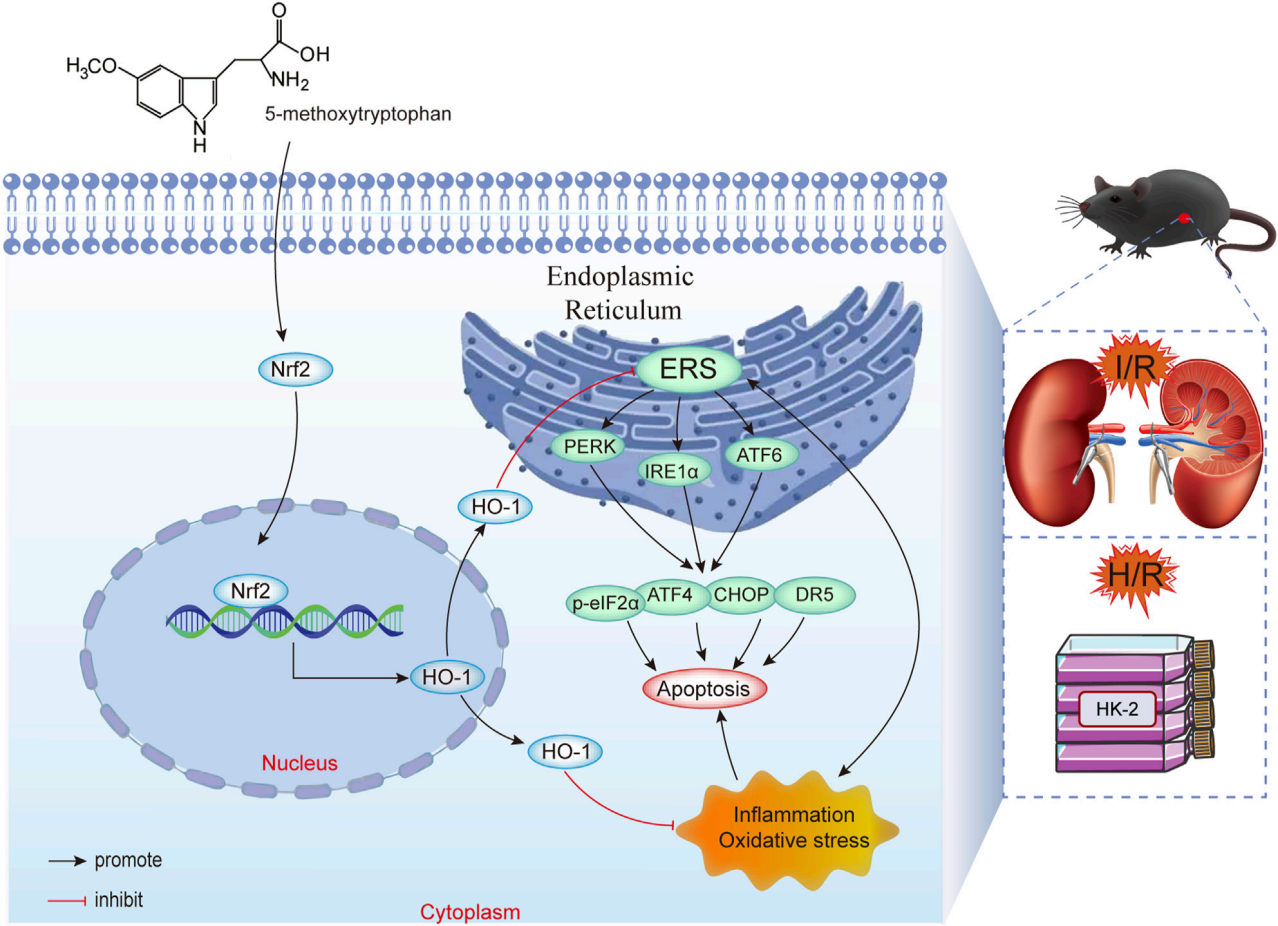
5-MTP mitigated renal I/R injury by reducing ERS-mediated apoptosis via the Nrf2/HO-1 pathway.

Our study confirmed that 5-MTP mitigates renal I/R injury by alleviating ERS-mediated apoptosis via the Nrf2/HO-1 pathway (Figure 7). First, we demonstrated that 5-MTP inhibited ERS-mediated apoptosis, thus alleviating renal injury *in vivo*. The experimental results revealed that 5-MTP could ameliorate inflammation, oxidative stress, and ERS-mediated apoptosis induced by H/R *in vitro*. The cytoprotective effect was suppressed in cells in which Nrf2 was silenced. Consistent with our *in vitro* findings, we observed partial abrogation of the therapeutic effect mediated by 5-MTP in Nrf2-KO mice. Thus, our finding improve the understanding of the organoprotective properties of 5-MTP and highlight its potential as a new therapeutic choice for renal I/R injury.

Renal I/R injury, characterized by damage to renal tissue and functional impairment, is a common clinical condition that may progress to chronic kidney disease (Yang et al., 2023). Through clamping of the bilateral renal pedicles, we developed an *in vivo* model that precisely mimics the essential characteristics of renal I/R injury. These characteristics include severe renal tissue damage, declined renal function, and elevated inflammation and oxidative stress. To elucidate the underlying mechanisms underlying the renoprotective effect of 5-MTP, we performed *in vitro* experiments using HK-2 cells exposed to H/R.

The endogenic metabolite, 5-MTP, derived from tryptophan, is synthesized in various cells, and its synthesis is regulated by tryptophan hydroxylase (Cheng et al., 2012). Some studies have reported that 5-MTP exerts neuroprotective effects on spinal cord injury by inhibiting inflammation and fibrosis formation (Hong et al., 2020). Moreover, 5-MTP can alleviate myocardial cell damage after myocardial infarction by mitigating oxidative stress injury, regulating mitochondrial stability, inhibiting inflammation, and modulating immune stress (Hsu et al., 2021). Our findings confirm that in renal I/R injury, 5-MTP can decrease IL-1β and IL-6 levels, indicating that it can mitigate inflammatory responses. Additionally, the GSH/GSSG ratio and T-AOC levels confirm that 5-MTP can alleviate oxidative stress injury, which was consistent with its previously reported anti-inflammatory and antioxidant abilities. We also detected the mRNA levels of KIM-1 and NGAL, which are markers of kidney injury. Other researchers have shown that the level of KIM-1 serves as a specific and sensitive diagnostic indicator for acute kidney injury and a reliable prognostic marker (Tutunea-Fatan et al., 2024). Similarly, the level of NGAL in the serum and kidneys was found to reflect the severity of renal injury (Allegretti et al., 2020; Játiva et al., 2022). Our study demonstrate that 5-MTP can significantly reduce structural damage to renal tissue and notably decrease KIM-1 and NGAL mRNA expression, as shown through HE staining and mRNA level analysis. Additionally, by detecting the levels of renal function indicators, such as SCr and BUN, this study reveal that 5-MTP can protect renal function in I/R injury. Renal tissue damage and impaired kidney function are hallmarks of acute kidney injury resulting from I/R injury in clinical practice. Inflammation and oxidative stress play key roles in exacerbating this condition. Our findings reveal that 5-MTP can mitigate renal tissue damage, enhance kidney function, and suppress inflammation and oxidative stress in mice with renal I/R injury. These results imply that 5-MTP can be used for the clinical treatment of renal I/R injury.

The endoplasmic reticulum plays a primary role in the cellular processes of protein folding, assembly, modification, and transport (Hetz et al., 2020). Protein folding and modification are precise and complex processes regulated by multiple mechanisms. Under various pathological conditions, such as hypoxia-ischemia and calcium overload, protein folding and modification can be disrupted, leading to ERS (Deshwal et al., 2018; Nagar et al., 2023). ERS can activate pro-survival and pro-apoptotic signals simultaneously (Lu et al., 2017; Weissman et al., 2006). Moderate ERS promotes cell survival, whereas sustained or excessive ERS can induce cell apoptosis through multiple pathways. ERS regulates the transcription factor CHOP through ATF4, ultimately leading to the induction of several pro-apoptotic factors, such as DR5, which triggers apoptosis (Kasetti et al., 2020; Song et al., 2023). The PERK/eIF2α/ATF4/CHOP pathway has been demonstrated to play a crucial role in ERS-mediated apoptosis. Some studies have shown that inhibiting this pathway can considerably reduce apoptosis across multiple diseases (Guo et al., 2017; Yu et al., 2021; Chen et al., 2020). In this study, we first detected the levels of ERS-related proteins via Western blotting analysis and confirmed that 5-MTP ameliorated the excessive ERS caused by renal I/R injury. We also detected the protein levels of ATF4, CHOP, and DR5 and the phosphorylation levels of eIF2α. These results indicate that 5-MTP can alleviate cell apoptosis caused by ERS, probably by inhibiting the PERK/eIF2α/ATF4/CHOP pathway. The results of the TUNEL and flow cytometry assays showed that 5-MTP considerably mitigated the apoptosis induced by I/R injury. These findings indicated that 5-MTP can ameliorate renal I/R injury by alleviating ERS-mediated apoptosis *in vivo* and *in vitro*.

The transcription factor Nrf2 is ubiquitously expressed across multiple organ systems. It exerts anti-inflammatory and antioxidant stress effects by activating downstream factors, thereby modulating the progression of many diseases (Zhang et al., 2022b). The activation of the Nrf2/HO-1 pathway is associated with mechanisms that alleviate diseases through mechanisms, including regulation of mitochondrial homeostasis, suppression of ferroptosis, and modulation of ERS (Xin et al., 2024; Wang et al., 2023; Shi et al., 2023). Moreover, 5-MTP can activate the Nrf2/HO-1 pathway, thus alleviating renal tissue inflammation after ureteral obstruction, exerting protective effects on both kidney and renal function, and exerting antirenal fibrosis effects (Chen et al., 2019). Our study further demonstrated that Nrf2 deficiency significantly inhibited 5-MTP’s therapeutic effects, including reduction of inflammation and oxidative stress, alleviation of renal tissue damage and renal function impairment. Additionally, the regulation of ERS by 5-MTP and its improvement in ERS-mediated apoptosis were strongly suppressed. These results indicate that the Nrf2/HO-1 pathway is required for regulating ERS and its associated apoptosis by 5-MTP.

Our study reveal that 5-MTP exerts anti-inflammatory and antioxidant effects on renal I/R injury, mirroring the actions of *N*-acetylcysteine, a drug with well-established renoprotective effects. Although *N*-acetylcysteine can mitigate contrast-induced nephropathy, its clinical utility is limited by low bioavailability and a brief renal residence time (Zhang et al., 2021). Therefore, future studies should focus on examining the renal residence time of 5-MTP and developing biomaterials to increase its bioavailability and extend its duration of action in the kidney, which may help enhance the clinical application and therapeutic efficacy of 5-MTP in the treatment of renal I/R injury and other renal diseases. This study also investigated the role of 5-MTP in ERS-mediated apoptosis and inflammation in renal I/R injury through the Nrf2/HO-1 pathway. However, the pathogenesis of renal I/R injury is complex and involves multiple apoptotic and inflammatory pathways. Further research on the regulatory effects of 5-MTP on other apoptotic pathways, including the mitochondrial apoptotic pathway and death receptor-mediated apoptosis, may help elucidate its specific mechanism of action in modulating apoptosis. Moreover, examining the interaction between 5-MTP and other inflammatory signaling pathways, such as the NF-κB and Toll-like receptor pathways, may provide insights into its anti-inflammatory mechanisms.

While our study confirms that 5-MTP confers protection against renal I/R injury via activation of the Nrf2/HO-1 pathway, three key limitations must be acknowledged. First, our results reveal that 5-MTP significantly increases the nuclear-to-cytoplasmic ratio of Nrf2, supporting its role in promoting active nuclear translocation. Although cytoplasmic Nrf2 levels were elevated, the disproportionate nuclear enrichment suggests that 5-MTP enhances nuclear trafficking while simultaneously stabilizing total Nrf2 protein or promoting its synthesis. This dual mechanism indicates that Nrf2 activation may involve both translocation and protein-level regulation. However, future studies are needed to dissect the precise mechanisms underlying 5-MTP-driven nuclear import and transcriptional upregulation. Second, the 24-h observation window in our experimental design precludes evaluation of long-term histological and functional outcomes. Chronic injury models may require extended therapeutic paradigms involving repeated 5-MTP administration or delayed intervention. Additional temporal endpoints should be incorporated to systematically track fibrotic progression and glomerulotubular functional recovery. Finally, despite mechanistic evidence from *in vivo* and *in vitro* models, clinical translational studies are needed to validate endogenous protective mechanisms in humans.

## 5 Conclusion

This study demonstrated that 5-MTP mitigates renal I/R injury and its attenuation of ERS-mediated apoptosis is strongly associated with the activation of the Nrf2/HO-1 pathway. These results indicate that 5-MTP may serve as a new treatment choice for renal I/R injury.

## Data Availability

Publicly available datasets were analyzed in this study. This data can be found here: https://www.ncbi.nlm.nih.gov/geo/query/acc.cgi?acc&equals;GSE212678.
