# Supplementary material for: 5-methoxytryptophan ameliorates renal ischemia/reperfusion injury by alleviating endoplasmic reticulum stress-mediated apoptosis through the Nrf2/HO-1 pathway
Source: Front Pharmacol. 2025 Apr 14;16:1506482. doi: 10.3389/fphar.2025.1506482 (PMC12034636; doi:10.3389/fphar.2025.1506482)
Supplement: Supplementary file 1 [file DataSheet1.zip › Table/Table S1.docx]

Table S1 Primer Sequences

| Genes | Forward primer | Reverse primer |
| --- | --- | --- |
| *KIM-1* | CCTGCTGCTACTGCTCCTTGTG | CCTGCTGCTACTGCTCCTTGTG |
| *NGAL* | TGGCCCTGAGTGTCATGTG | CTCTTGTAGCTCATAGATGGTGC |
| *Gapdh* | AGGTCGGTGTGAACGGATTTG | TGTAGACCATGTAGTTGAGGTCA |
| *NRF2* | AACCACCCTGAAAGCACAGC | TGAAATGCCGGAGTCAGAATC |
| *GAPDH* | TCCAAAATCAAGTGGGGCGAT | TTCTAGACGGCAGG TCAGGTC |
